# Post-transplant lymphoproliferative disorder presenting as a tumor adjacent to the renal allograft: A case report and review of the literature

**DOI:** 10.3892/ol.2014.2586

**Published:** 2014-10-02

**Authors:** CHEN GAO, LONGKAI PENG, FENGHUA PENG, TING TUO, DAIQIANG LI

**Affiliations:** 1 Department of Urological Organ Transplantation, Second Xiangya Hospital, Central South University, Changsha, Hunan 41011, P.R. China; 2 Department of Pathology, Second Xiangya Hospital, Central South University, Changsha, Hunan 41011, P.R. China

**Keywords:** post-transplant lymphoproliferative disorder, Epstein-Barr virus, early-onset, kidney transplantation

## Abstract

Post-transplant lymphoproliferative disorder (PTLD) is a potentially fatal complication of solid organ transplantation. The current report presents the case of a 42-year-old male who developed PTLD within the first year following renal transplantation. The disorder manifested as a tumor adjacent to the lower pole of the renal allograft and resulted in urinary obstruction. Durable complete remission was achieved as a result of surgical resection followed by a reduction in immunosuppression and low-dose rituximab-based chemotherapy, indicating that this therapeutic strategy may be safe and effective for the treatment of specific cases of localized and resectable PTLD.

## Introduction

Post-transplant lymphoproliferative disorder (PTLD) is a rare, but critical complication that occurs following solid organ and hematopoietic stem cell transplantation (1). PTLD encompasses a heterogeneous group of disorders, ranging from benign self-limited lesions to aggressive widely disseminated disease. Generally, PTLD is considered to be an iatrogenic complication due to the intensive immunosuppressive treatment administered following transplantation (2). The overall incidence of PTLD in adult kidney transplantation surgery was between 1 and 3% worldwide (3). However, early-onset PTLD (<1 year from transplantation to presentation of PTLD) is closely associated with the Epstein-Barr virus (EBV) infection and exhibits a predilection for allograft localization (4), occasionally occuring at sites adjacent to the allograft. The current study describes a rare case of PTLD that presented as a tumor adjacent to the allograft within the first year following renal transplantation. The treatment strategy included surgical resection that was followed by a reduction in immunosuppression and low-dose rituximab-based chemotherapy. This study may lead to future improvements for the treatment of post-transplant lymphoproliferative disorder. Written informed consent was obtained from the patient.

## Case report

**Figure 1:**
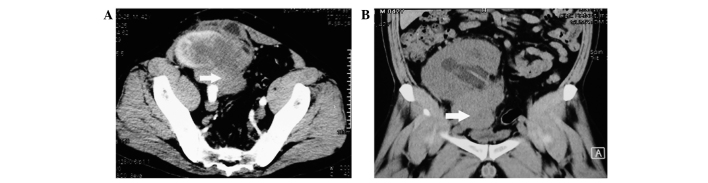
(A) Abdominal computed tomography showing a solid mass adjacent to the renal allograft. (B) Abdominal computed tomography with coronal multiplanar reformation showing a solid mass adjacent to the lower pole of the renal allograft and hydronephrosis. The arrow indicates the solid mass.

**Figure 2:**
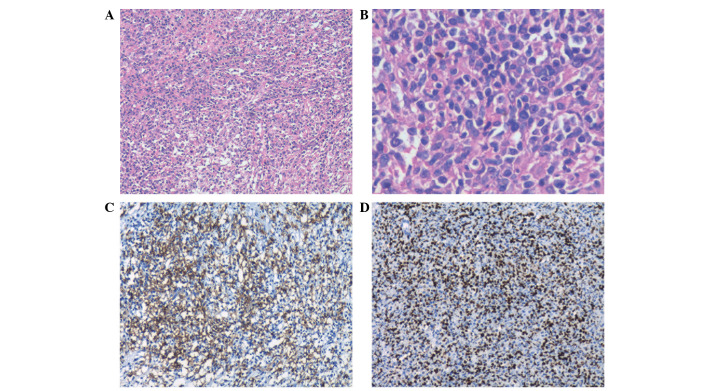
Histopathological images of the tumor. (A and B) Diffuse infiltration of lymphocytes, immunoblasts and plasma cells (stain, hematoxylin and eosin). Abundant lymphoid cells were positive for (C) cluster of differentiation 20 (staining, brown) and (D) Epstein-Barr virus-encoded RNA *in situ* hybridization (staining, brown). Magnification, (A, C and D) ×100; (B) ×400.

In March 2012, a 42-year-old male exhibiting end-stage renal disease secondary to hypertension received a kidney transplant at the Second Xiangya Hospital of Central South University (Changsha, China). The donor was a 27-year-old male who had succumbed to cardiac failure caused by craniocerebral trauma. The human leukocyte antigen (HLA) type of the donor and the recipient were A11/A2-B58/B13-DR4/DR53, DR9/DR53 and A2/A2-B13/B61-DR15/DR51, DR9/DR53, respectively. The recipient was EBV seronegative, however, the donor’s EBV serologic status was unknown, as EBV serologic status was not tested routinely at the time of donation. Furthermore, the donor and recipient were seronegative for cytomegalovirus, and the Hepatitis B and C viruses. There were no complications during surgical follow-up. The patient’s post-transplant immunosuppressive regimen included: Intravenous methylprednisolone (dose during surgery, 0.5 g; dose for the first three days following surgery, 0.5 g/day); followed by cyclosporine (CsA; initial dose, 6 mg/kg/day; trough concentration was adjusted to 220–250 ng/ml 0–6 months following transplantation and 180–220 ng/ml over the next 6–12 months); oral mycophenolate mofetil (MMF; dose, 0.75 g per 12 h; gradually reduced to 0.5 g per 12 h for long-term maintenance immunosuppression); and prednisolone (initial dose, 80 mg/day; gradually reduced to 10 mg/day over the next 6 months for long-term maintenance immunosuppression). Good renal allograft function was observed immediately following surgery. On the twelfth postoperative day, the patient exhibited a serum creatinine level of 133 μmol/l (normal range, 44–133 μmol/l) and was discharged. The allograft function remained normal during the out-patient follow-up, however, seven months post-transplantation, the patient developed a fever, oliguria and an elevated serum creatinine level (410.7 μmol/l). Sonography and computed tomography revealed a solid mass adjacent to the renal allograft (Fig. 1A) and computed tomography with coronal multiplanar reformation revealed a solid mass (size, 6×4×8 cm) in the lower pole of the allograft and hydronephrosis (Fig. 1B). Percutaneous nephrostomy tubes were inserted, resulting in a decline in the serum creatinine level. Subsequently, surgical resection was performed and a tumor with a poorly defined margin, located adjacent and in close proximity to the lower pole of the allograft, was removed. Postoperatively, serum creatinine returned to within the normal range following treatment of the urinary obstruction. Intra- and postoperative histopathological assessments determined a diagnosis of polymorphic PTLD with positive stains for cluster of differentiation (CD)20, CD79a, CD3 and EBV-encoded RNA (Fig. 2). The proliferation index of Ki-67 was 40%. Upon diagnosis, the blood EBV DNA level was 1.3×10^4^ copies/ml, and the lactate dehydrogenase level (normal range, 135–215 U/l) and bone marrow biopsy were normal. Therefore, MMF therapy was discontinued and CsA was replaced with Rapamune (Wyeth Pharmaceuticals, Dallas, TX, USA) (trough concentration adjusted to 6–8 ng/ml) to reduce the level of immunosuppression. Furthermore, four cycles of adjuvant low-dose chemotherapy were administered, including rituximab (300 mg/m^2^), cyclophosphamide (500 mg/m^2^), vincristine (1.2 mg/m^2^) and prednisolone (50 mg/m^2^). The patient’s blood was negative for EBV DNA following the first cycle of chemotherapy. During the 16-month follow-up after resection, the patient remained in remission, neither EBV viremia nor PTLD recurred and renal allograft function was preserved. Outpatient follow-up is ongoing to determine the long-term outcome of the treatment strategy.

## Discussion

PTLD is the second most commonly occurring malignancy in solid organ transplant recipients, worldwide. Approximately 20% of kidney transplant patients developed PTLD within the first year following surgery (5). Early-onset PTLD occurs more commonly in EBV seronegative recipients compared with EBV seropositive recipients and is characterized by an EBV *in situ* hybridization-positive, CD20-positive phenotype and allograft involvement (6). An immunosuppressed state and EBV infection are considered to be the two most important risk factors in PTLD development (2). In the majority of EBV-associated cases of PTLD, immunosuppression depresses the EBV-specific cellular immune response, which may promote uncontrolled EBV-infected lymphocyte proliferation, resulting in PTLD (7). The rare case described in the current report presented as a tumor adjacent to the lower pole of the renal allograft and developed into a urinary obstruction. To the best of our knowledge, few similar cases have been reported to date (8–12). In the present case, the risk factors for the development of PTLD included EBV seronegativity prior to transplantation, EBV infection post-transplantation, mismatching at the HLA-B locus and a high dose of CsA (13,14).

No consensus on the optimal management of PTLD has been determined, however, a reduction in immunosuppression (RI) has been demonstrated to be an effective initial treatment modality for PTLD. In a recent analysis of 148 solid organ transplant-associated PTLD cases, Reshef *et al* (15) reported that the overall response rate for a RI alone was 45% and the three year overall survival rate was 55%. Recent guidelines recommend commencing the reduction of immunosuppression therapy as soon as possible in all PTLD patients (16) and in specific cases of localized PTLD, surgical excision of isolated lesions or debulking of the tumor may be an effective component of first-line treatment. Reshef *et al* (15) identified that patients who underwent surgery and adjuvant RI exhibited a favorable outcome, with 27% patients relapsing at a median of five months. Rituximab is a monoclonal antibody against the B lymphocyte-specific CD20 antigen (17). Recent data indicates that immediate commencement of rituximab-based therapy followed by anthracycline-based chemotherapy (cyclophosphamide, doxorubicin, vincristine and prednisolone) may result in durable progressive-free survival in PTLD patients (18) and may reduce the risk of renal graft impairment following the reduction of immunosuppression (19). In the present case, surgical excision was performed for the diagnosis and resolution of the urinary obstruction. RI, followed by rituximab-based therapy combined with low-dose chemotherapy, for four months, the standard dose is as follows: rituximab (375 mg/m^2^, day 1), cyclophosphamide (400 mg/m^2^, days 1–5), vincristine (1.4 mg/m^2^, day 1) and prednisolone (100 mg/m^2^, days 1–5) and was prescribed due to the aggressive nature of the disease. The therapeutic strategy administered to the patient in the present study resulted in complete remission with few manageable side-effects, including nausea/vomiting and leukopenia. Consistent with the present case, two previous studies reported that surgical intervention in combination with other therapies achieved durable remission in specific patients exhibiting localized PTLD (20,21). The maintenance of immunosuppression remains a challenge in renal recipients who develop PTLD. The use of calcineurin inhibitors has been associated with an increased incidence of PTLD (22), however, treatment with rapamycin and its analogs has demonstrated immunosuppression and antiproliferative action. Previous studies demonstrated that rapamycin immunosuppressant therapy produced favorable effects in kidney transplant patients exhibiting PTLD (23,24). Therefore, serolimus was introduced in the present case for maintenance immunosuppression. However, the use of rapamycin in PTLD patients remains controversial (25), thus, further studies are required to reach a consensus for the optimal management of PTLD.

In conclusion, the current report presents a rare case of PTLD that manifested as an obstructive uropathy within the first year following kidney transplantation. Surgical excision followed by a reduction in immunosuppression and low-dose rituximab-based chemotherapy may present as an effective and safe strategy for specific cases of localized and resectable PTLD.
